# Astragaloside IV Inhibits NF-***κ***B Activation and Inflammatory Gene Expression in LPS-Treated Mice

**DOI:** 10.1155/2015/274314

**Published:** 2015-04-16

**Authors:** Wei-Jian Zhang, Balz Frei

**Affiliations:** Linus Pauling Institute and Department of Biochemistry and Biophysics, Oregon State University, 307 Linus Pauling Science Center, Corvallis, OR 97331, USA

## Abstract

In this study we investigated the role of astragaloside IV (AS-IV), one of the major active constituents purified from the Chinese medicinal herb *Astragalus membranaceus*, in LPS-induced acute inflammatory responses in mice *in vivo* and examined possible underlying mechanisms. Mice were assigned to four groups: vehicle-treated control animals; AS-IV-treated animals (10 mg/kg b.w. AS-IV daily i.p. injection for 6 days); LPS-treated animals; and AS-IV plus LPS-treated animals. We found that AS-IV treatment significantly inhibited LPS-induced increases in serum levels of MCP-1 and TNF by 82% and 49%, respectively. AS-IV also inhibited LPS-induced upregulation of inflammatory gene expression in different organs. Lung mRNA levels of cellular adhesion molecules, MCP-1, TNF*α*, IL-6, and TLR4 were significantly attenuated, and lung neutrophil infiltration and activation were strongly inhibited, as reflected by decreased myeloperoxidase content, when the mice were pretreated with AS-IV. Similar results were observed in heart, aorta, kidney, and liver. Furthermore, AS-IV significantly suppressed LPS-induced NF-*κ*B and AP-1 DNA-binding activities in lung and heart. In conclusion, our data provide new *in vivo* evidence that AS-IV effectively inhibits LPS-induced acute inflammatory responses by modulating NF-*κ*B and AP-1 signaling pathways. Our results suggest that AS-IV may be useful for the prevention or treatment of inflammatory diseases.

## 1. Introduction

Endothelial cells are a primary target of inflammatory responses, and their injury can lead to vasculopathy and organ dysfunction [1]. The bacterial endotoxin LPS directly elicits several acute inflammatory responses in endothelial cells, including production of cellular adhesion molecules, such as E-selectin, vascular cell adhesion molecule-1 (VCAM-1), and intercellular adhesion molecule-1 (ICAM-1), and other proinflammatory mediators, such as TNF*α*, IL-6, and monocyte chemoattractant protein-1 (MCP-1). Together, these proinflammatory mediators elicit leukocyte adhesion to the vasculature and transmigration into the underlying tissue, causing endothelial injury and dysfunction associated with sepsis [1]. Although these proinflammatory mediators are required for an adequate host-defense response, their dysregulation can lead to refractory hypotension, cardiovascular hyporeactivity, intravascular coagulation, multiple organ failure, and death [2, 3].

The regulation of inflammatory gene transcription has been shown to be controlled by specific signaling pathways and transcription factors, such as NF-*κ*B and AP-1 [4, 5]. In particular, the NF-*κ*B pathway affects host defense against infectious agents by upregulating inflammatory genes that cause acute neutrophilic inflammation and the systemic inflammatory response syndrome [5]. Under normal condition, NF-*κ*B is located in the cytoplasm in an inactive form in association with its inhibitor, I*κ*B. In response to stimulation, for example, by LPS, I*κ*B is phosphorylated by I*κ*B kinase. Following phosphorylation, I*κ*B is ubiquitinated and degraded, allowing NF-*κ*B to translocate to the nucleus, bind to DNA promoter regions, and induce inflammatory gene transcription. Various agents that block NF-*κ*B signaling have been shown to decrease expression of proinflammatory mediators [6–9]. Thus, the inhibition of NF-*κ*B activation is expected to be protective in pathological inflammatory states.

*Astragalus membranaceus* is one of the most widely used Chinese medicinal herbs for the treatment of many diseases, including cardiovascular disease, nephritis, hepatitis, and diabetes [10]. It also has been available in Europe and the US for many years as a food supplement. Evidence from pharmacological research and clinical practice suggests that* Astragalus* possesses a wide spectrum of activities, including immunomodulation [11, 12], cardiovascular protection [13–15], anti-inflammatory effects [16–18], hepatoprotection [19, 20], antidiabetes [21], anticancer [22], and neuroprotection [23].

The biologically active constituents of* Astragalus* roots represent three classes of chemical compounds: saponins, polysaccharides, and flavonoids [24]. By chemical degradation and ^13^C nuclear magnetic resonance examination, the structure of astragaloside IV (AS-IV) was determined as 3-O-*β*-D-xylopyranosyl-6-O-*β*-D-glucopyranosyl-cycloastragenol (C_41_H_68_O_14_; MW = 784.9) [25]. As one of the major active constituents of* Astragalus*, AS-IV is used as the characteristic marker for quality evaluation of* Astragalus* in the Chinese Pharmacopeia and has been shown to exert potent cardioprotective and anti-inflammatory effects [6, 10, 26–30]. We have previously shown that AS-IV inhibits LPS- and TNF*α*-induced adhesion molecule expression and NF-*κ*B activation in cultured human endothelial cells [6]. However,* in vivo* evidence supporting such activity is currently lacking. Therefore, in this study we investigated whether AS-IV can inhibit LPS-induced acute inflammatory responses in experimental mice.

## 2. Materials and Methods

### 2.1. Animals and Experimental Procedures

Female C57BL/6J mice, 12 weeks old and weighing 20–22 g, were purchased from Jackson Laboratory (Bar Harbor, ME) and housed in specific pathogen-free conditions and a temperature- and humidity-controlled environment (12-h light/dark cycle) with unlimited access to tap water and Purina 5001 chow diet (Harlan Teklad, Madison, WI). The investigation conformed to the* Guide for the Care and Use of Laboratory Animals* by NIH, and all animal procedures were reviewed and approved by the Oregon State University Institutional Animal Care and Use Committee.

AS-IV was purchased from Quality Phytochemicals LLC (Edison, NJ), and a stock solution was prepared with propylene glycol (Sigma Aldrich, St. Louis, MO) and further diluted with Hank's buffered saline solution (HBSS). LPS (serotype 055:B5 from* Escherichia coli, *Sigma Aldrich) stock solution was prepared in HBSS. Mice were randomly assigned to 4 groups as follows: (i) control animals received daily i.p. injection of the vehicle propylene glycol with HBSS for 6 days followed by a single i.p. HBSS injection; (ii) AS-IV-treated animals received 10 mg/kg b.w. AS-IV daily i.p. for 6 days followed by a single i.p. HBSS injection; (iii) LPS-treated animals received daily i.p. injection of propylene glycol with HBSS for 6 days followed by a single i.p. injection of 0.5 *μ*g/g b.w. LPS; and (iv) AS-IV plus LPS-treated animals received AS-IV daily i.p. for 6 days followed by single i.p. injection of LPS. Animals were sacrificed 3 hours after HBSS or LPS injection. Based on our previous observations, the 3-h time point and LPS dose of 0.5 *μ*g/g b.w. were chosen [31]. In some studies, mice were randomly assigned to receive i.p. injection of LPS and sacrificed after 1, 3, 8, or 24 h. Each group consisted of 4 to 5 animals. After sacrifice, blood and tissues were collected for further analysis.

### 2.2. Serum Inflammatory Mediators

Serum concentrations of MCP-1, TNF*α*, sVCAM-1, and sICAM-1 were measured by quantitative colorimetric sandwich ELISA (R&D Systems, Minneapolis, MN). The sensitivity of the assays is 2 pg/mL for MCP-1, 5 pg/mL for TNF*α*, and 30 pg/mL for sVCAM-1 and sICAM-1.

### 2.3. Tissue mRNA Levels of Inflammatory Mediators

Total RNA was isolated from different organs using TRIzol Reagent (Life Technologies, Foster City, CA). cDNA synthesis was performed using the high capacity cDNA archive kit (Life Technologies). mRNA levels of* VCAM-1, ICAM-1, E-selectin, P-selectin, MCP-1, TNFα, IL-6*, myeloperoxidase* (MPO)*, Toll-like receptor-4* (TLR4)*, and glyceraldehyde-3-phosphate dehydrogenase* (GAPDH)* were quantitated by real-time qPCR. All primers and probes were purchased as kits (Assays on Demand, Life Technologies). The assays are supplied as a 20x mixture of PCR primers and TaqMan minor groove binder 6-FAM dye-labeled probes with a nonfluorescent quencher at the 3′ end. TaqMan quantitative PCR (40 cycles at 95°C for 15 sec and 60°C for 1 min) was performed using TaqMan Universal PCR Master Mix (Life Technologies) in 96-well plates with an ABI Prism 7500 Sequence Detection System (Life Technologies). To obtain relative quantification, two standard curves were constructed in each plate with one target gene and the internal control* GAPDH* gene. Standard curves were generated by plotting the threshold cycle number values against the log of the amount of input cDNA and used to quantify the expression of the target genes and* GAPDH* gene in the same sample. After normalization to internal* GAPDH* in each sample, results were expressed as percentage of GAPDH or fold of control.

### 2.4. Lung Protein Concentration of Myeloperoxidase

A part of the right lung lobe was homogenized, and the cytosolic fraction of lung tissue homogenate was prepared using nuclear extract kits (Active Motif, Carlsbad, CA). Lung cytosolic MPO was quantified using the MPO enzyme-linked immunosorbent assay kit (Hycult Biotechnology, Plymouth Meeting, PA) according to the manufacturer's instructions. The lung MPO concentration of each sample was normalized with cytosolic protein concentration and expressed as ng MPO/mg tissue protein.

### 2.5. Nuclear Transcription Factors

Nuclear extracts were prepared from lung and heart using nuclear extract kits (Active Motif). For analysis of nuclear transcription factor activation, ELISA-based assays (Active Motif) were used to determine the DNA-binding activity of NF-*κ*B (p65) and AP-1 (c-fos). The specificity of binding was confirmed by competition with either wild-type or mutant oligonucleotides.

### 2.6. Statistical Analysis

The data were calculated as means ± SEM and analyzed by ANOVA with Fisher PLSD post hoc test. Statistical significance was set at *P* < 0.05.

## 3. Results and Discussion

The pathophysiology of acute inflammation triggered by the bacterial endotoxin LPS is characterized by the production of multiple proinflammatory cytokines and chemokines, expression of adhesion molecules, and infiltration of neutrophils and monocytes into inflamed tissues. Because of the complexity of the pathology of septic shock, major efforts have focused on identifying novel anti-inflammatory drugs that prevent the proinflammatory process at the early stage of gene expression of key inflammatory mediators [32].

We have previously shown that AS-IV inhibits LPS- and TNF*α*-induced adhesion molecule expression and consequent adherence of monocytes by modulating NF-*κ*B signaling pathway in cultured human endothelial cells [6]. We now provide new evidence showing that AS-IV exhibits strong anti-inflammatory activities* in vivo* by attenuating LPS-induced acute inflammatory responses through inhibition of NF-*κ*B- and AP-1-mediated inflammatory signaling pathways in mice.

### 3.1. AS-IV Inhibits LPS-Induced Increases in Serum MCP-1 and TNF*α* in Mice

**Figure 1:**
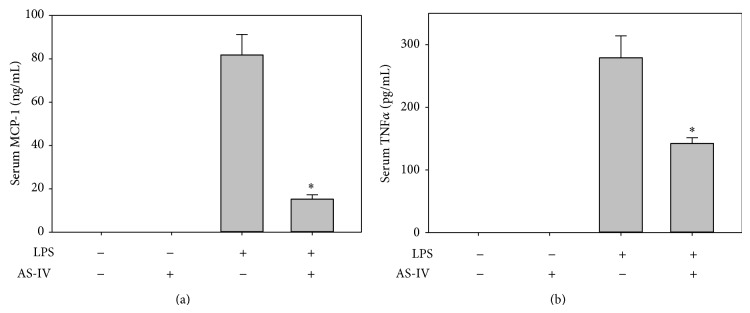
AS-IV inhibits LPS-induced increases in serum MCP-1 and TNF*α* in mice. Mice were randomly assigned to 4 groups as follows: (i) control animals received daily i.p. injection of the vehicle HBSS for 6 days followed by a single i.p. HBSS injection; (ii) AS-IV-treated animals received 10 mg/kg b.w. AS-IV daily i.p. for 6 days followed by a single i.p. HBSS injection; (iii) LPS-treated animals received daily i.p. injection of vehicle HBSS for 6 days followed by a single i.p. injection of 0.5 *μ*g/g b.w. LPS; and (iv) AS-IV plus LPS-treated animals received AS-IV daily i.p. for 6 days followed by single i.p. injection of LPS. Three hours after the HBSS or LPS injection, the animals were sacrificed and blood was collected. Serum MCP-1 (a) and TNF*α* (b) were measured by ELISA. Data shown are mean values ± SEM of five animals per group. ^∗^ *P* < 0.05 compared to animals treated with LPS only.

Treatment of mice with AS-IV for 6 days had no effect on body weight changes compared to HBSS-treated control animals. The mean body weight was 20.8 ± 0.6 g before and 20.8 ± 0.5 g after 6-day AS-IV treatment. In control animals, the mean body weight was 20.2 ± 0.3 g before and 19.9 ± 0.4 g after 6-day HBSS treatment. We first investigated the effect of AS-IV on LPS-induced systemic inflammatory responses by determining serum levels of MCP-1, TNF*α*, and the soluble cellular adhesion molecules, sVCAM-1 and sICAM-1. Treatment of mice with AS-IV alone for 6 days had no effect on serum levels of these inflammatory mediators compared to HBSS-treated control animals. As expected, 3 hours after LPS injection, significant increases in the serum levels of MCP-1 and TNF*α* were observed (Figure 1). Interestingly, pretreatment of animals with AS-IV significantly inhibited LPS-induced increases in serum MCP-1 (Figure 1(a)) and TNF*α* (Figure 1(b)) by 82% and 49%, respectively. Specifically, MCP-1 levels were 15.2 ± 2.0 ng/mL in mice treated with AS-IV plus LPS, compared to 81.8 ± 9.5 ng/mL in animals treated with LPS only; TNF*α* levels were 142 ± 9 pg/mL and 279 ± 35 pg/mL, respectively (*P* < 0.05, *n* = 5). However, AS-IV did not inhibit the LPS-induced increases in serum sVCAM-1 and sICAM-1 concentrations (data not shown).

### 3.2. AS-IV Inhibits LPS-Induced Upregulation of Inflammatory Gene Expression in Mouse Lung and Other Tissues

**Figure 2:**
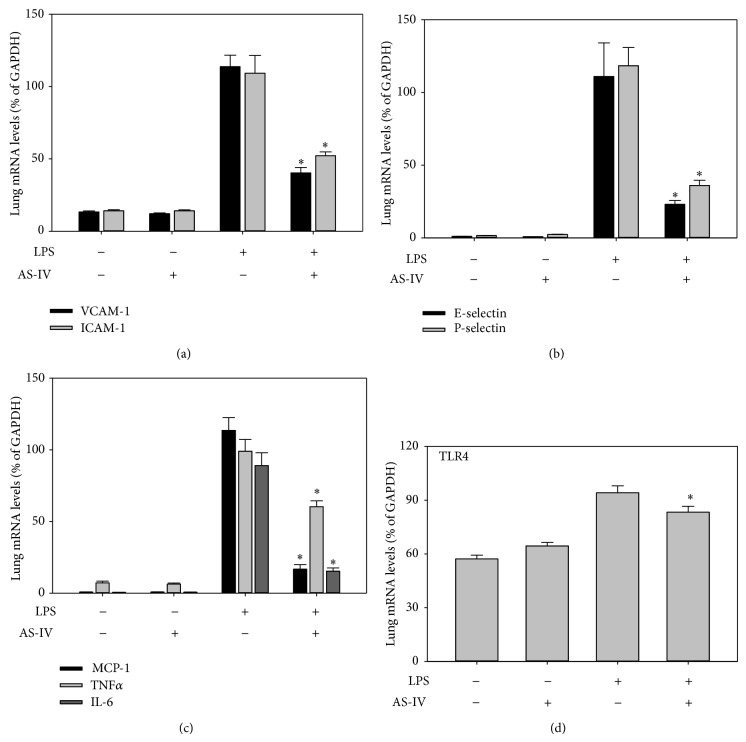
AS-IV inhibits LPS-induced inflammatory gene expression in mouse lung. Mice were randomly assigned to 4 groups as follows: (i) control animals received daily i.p. injection of the vehicle HBSS for 6 days followed by a single i.p. HBSS injection; (ii) AS-IV-treated animals received 10 mg/kg b.w. AS-IV daily i.p. for 6 days followed by a single i.p. HBSS injection; (iii) LPS-treated animals received daily i.p. injection of vehicle HBSS for 6 days followed by a single i.p. injection of 0.5 *μ*g/g b.w. LPS; and (iv) AS-IV plus LPS-treated animals received AS-IV daily i.p. for 6 days followed by single i.p. injection of LPS. Three hours after the HBSS or LPS injection, the animals were sacrificed and tissues were collected. Total RNA was isolated from lung. Inflammatory gene expression was quantified using real-time quantitative PCR. After normalization to the internal control gene GAPDH, the results for each target gene were expressed as percentage of GAPDH. Data shown are means ± SEM of 5 animals per group. ^∗^ *P* < 0.05 compared to animals treated with LPS only.

**Figure 3:**
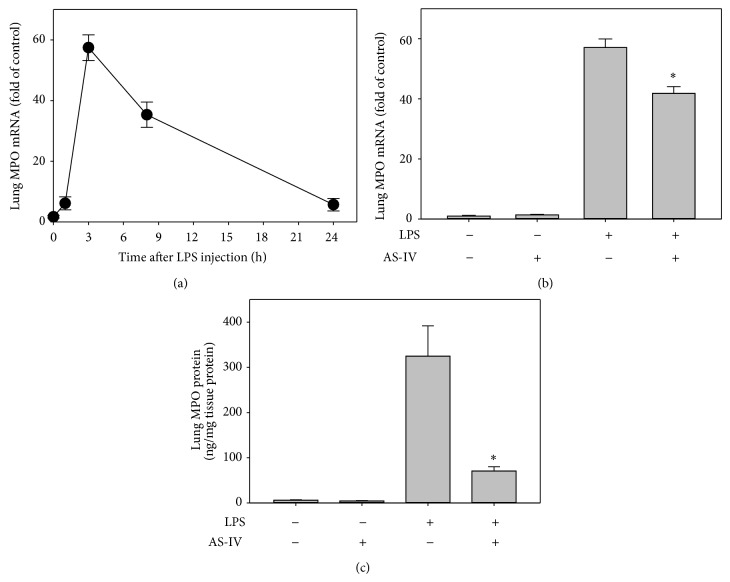
AS-IV inhibits LPS-induced gene and protein expression of myeloperoxidase (MPO) in mouse lung. For panel (a), mice were randomly assigned to receive i.p. injection of LPS and sacrificed after 1, 3, 8, or 24 h. For panels (b) and (c), mice were treated as described in the legend of Figure 2. Total RNA and cytosolic protein were isolated from lung. Lung* MPO* gene expression was quantified as described in the legend of Figure 2. Lung MPO protein was determined using an ELISA kit as described in detail in Section 2. The mRNA data are shown as fold of control after normalization to the internal control gene GAPDH. Data shown are means ± SEM of 4 to 5 animals per group. ^∗^ *P* < 0.05 compared to animals treated with LPS only.

**Figure 4:**
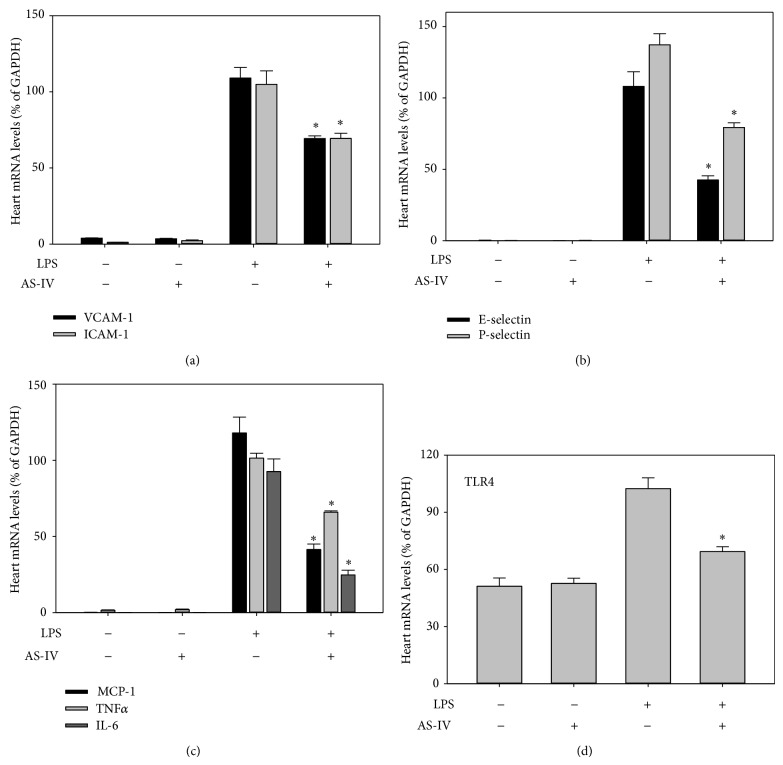
AS-IV inhibits LPS-induced inflammatory gene expression in mouse heart. Mice were treated as described in the legend of Figure 2. Total RNA was isolated from heart, and inflammatory gene expression was quantified as described in the legend of Figure 2. Data shown are means ± SEM of 5 animals per group. ^∗^ *P* < 0.05 compared to animals treated with LPS only.

**Figure 5:**
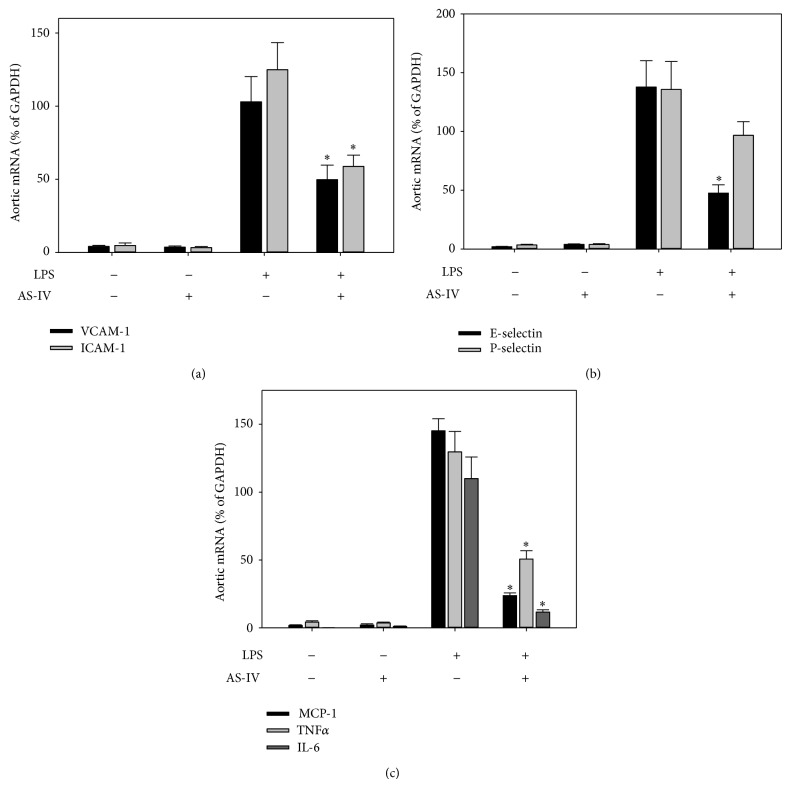
AS-IV inhibits LPS-induced inflammatory gene expression in mouse aorta. Mice were treated as described in the legend of Figure 2. Total RNA was isolated from aorta, and inflammatory gene expression was quantified as described in the legend of Figure 2. Data shown are means ± SEM of 5 animals per group. ^∗^ *P* < 0.05 compared to animals treated with LPS only.

**Figure 6:**
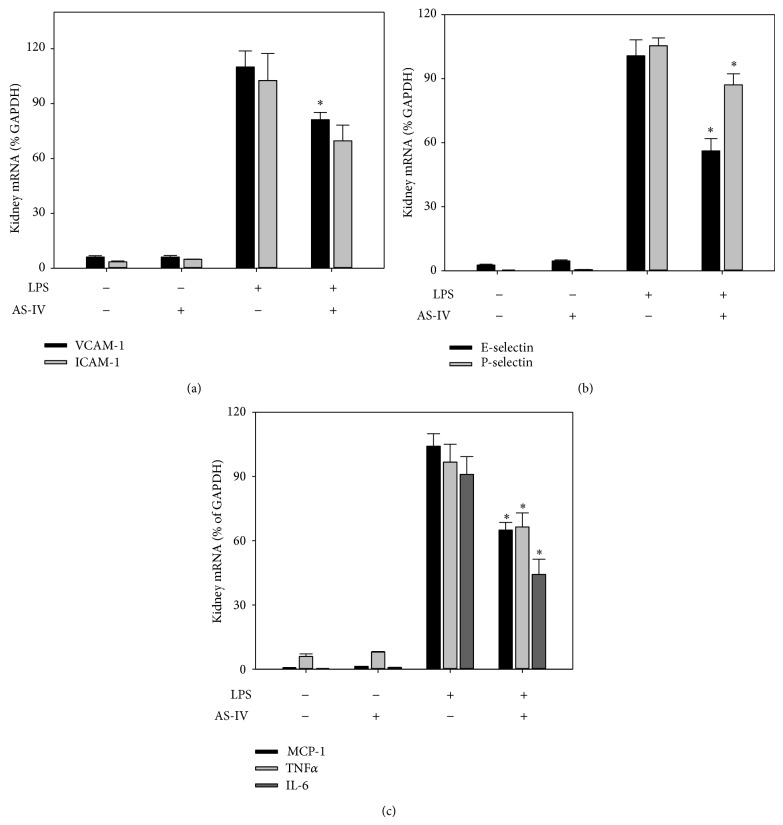
AS-IV inhibits LPS-induced inflammatory gene expression in mouse kidney. Mice were treated as described in the legend of Figure 2. Total RNA was isolated from kidney, and inflammatory gene expression was quantified as described in the legend of Figure 2. Data shown are means ± SEM of 5 animals per group. ^∗^ *P* < 0.05 compared to animals treated with LPS only.

To investigate whether AS-IV inhibits LPS-induced acute inflammatory responses in mouse organs, we assessed gene expression of inflammatory mediators in lung, heart, aorta, kidney, and liver of LPS-exposed mice, using real-time qPCR analysis. Treatment of mice with AS-IV alone did not affect gene expression of cellular adhesion molecules and proinflammatory mediators (Figures 2–6). As expected, treatment of mice with LPS for 3 hours strongly upregulated inflammatory gene expression in all tissues examined (Figures 2–6). Pretreatment of mice with AS-IV significantly inhibited the LPS-induced increase in lung mRNA levels of adhesion molecules:* VACM-1* by 73%,* ICAM-1* by 60%,* E-selectin* by 79%, and* P-selectin* by 71% and other proinflammatory mediators:* MCP-1* by 85%,* TNFα* by 42%,* IL-6* by 83%, and* TLR4* by 30% (*P* < 0.05, *n* = 5) (Figure 2). Similar inhibitory effects of AS-IV were also observed in heart (Figure 4), aorta (Figure 5), kidney (Figure 6), and liver (data not shown).

### 3.3. AS-IV Inhibits LPS-Induced Neutrophil Infiltration and Activation in Lung

As polymorphonuclear neutrophils (PMN) and other phagocytic cells, such as monocyte-macrophages, play critical roles in acute inflammation and tissue injury, we assessed lung myeloperoxidase (MPO), a well-documented PMN-specific biomarker [31, 33, 34], after LPS challenge. MPO is rapidly released when PMN are activated, which triggers transcriptional upregulation of mRNA and new protein synthesis of MPO. Therefore, upregulation of* MPO* mRNA levels also reflects PMN activation during acute lung inflammatory responses [31, 33]. As shown in Figure 3(a), lung* MPO* mRNA was at very low levels in control animals; however, LPS treatment induced time-dependent upregulation of lung* MPO* gene expression, which peaked at 3 h and remained elevated for up to 8 h and then declined after 24 h. These data indicate infiltration and activation of interstitial PMN in the lungs of LPS-treated mice in a time-dependent manner, which is consistent with our previous results showing that TNF*α* treatment induced increases in MPO mRNA and protein levels, enzyme activity, and morphological accumulation of PMN in mouse lung tissues [33]. However, pretreatment of mice with AS-IV significantly inhibited the LPS-induced increase in* MPO* mRNA level by 27% (*P* < 0.05, *n* = 5) (Figure 3(b)). This was further confirmed by the lung MPO protein level, which increased from 6 ± 1 ng/mg tissue protein in control animals to 325 ± 67 ng/mg tissue protein in LPS-treated animals and significantly reduced by AS-IV treatment by 80% to 71 ± 9 ng/mg tissue protein (*P* < 0.05, *n* = 5) (Figure 3(c)). As the lung is the main target for activated PMN during acute inflammation [31, 33, 34], these data support the notion that AS-IV inhibits PMN infiltration/recruitment to the lung and subsequent tissue damage during LPS-induced acute inflammatory responses.

### 3.4. AS-IV Inhibits LPS-Induced NF-*κ*B and AP-1 DNA-Binding Activity in Mouse Lung and Heart

**Figure 7:**
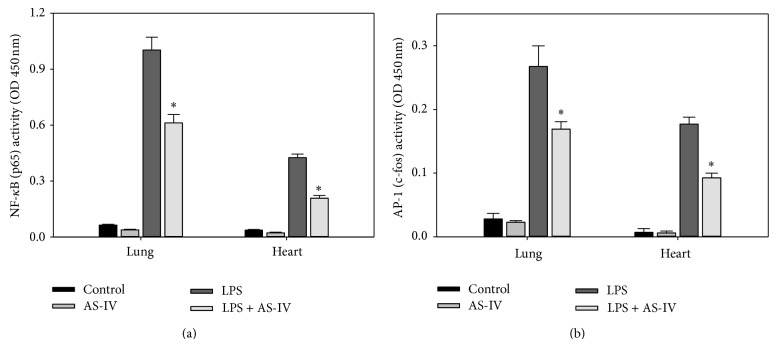
AS-IV inhibits LPS-induced NF-*κ*B (a) and AP-1 (b) DNA-binding activity in mouse lung and heart. Mice were treated as described in the legend of Figure 2. Nuclear extracts were isolated from lung and heart. DNA-binding activity of NF-*κ*B (p65) and AP-1 (c-fos) was quantified by ELISA. Data shown are means ± SEM of 5 animals per group. ^∗^ *P* < 0.05 compared to animals treated with LPS only.

It is well recognized that NF-*κ*B plays a prominent role in LPS-induced transcriptional regulation of most inflammatory genes that contribute to the development of septic shock, multiple organ failure, and death [5, 32]. Of clinical relevance, NF-*κ*B activation was increased in patients with acute inflammation and sepsis and correlated with clinical severity and mortality [35]. To investigate possible signaling pathways mediating the inhibitory effect of AS-IV on LPS-induced inflammatory gene transcription, we assessed the nuclear content of the NF-*κ*B subunit, p65, and the AP-1 subunit, c-fos, as indicators of nuclear translocation and activation of these transcription factors. The DNA-binding activity of NF-*κ*B (p65) and AP-1 (c-fos) was detectable at low levels in lung and heart tissues of control and AS-IV-only-treated animals. AS-IV alone did not cause NF-*κ*B or AP-1 activation in either lung or heart (Figure 7). LPS markedly increased NF-*κ*B activity in lung and heart by 15.8- and 11.5-fold, respectively; AS-IV treatment significantly suppressed LPS-induced activation of NF-*κ*B by 42% and 54%, respectively (*P* < 0.05, *n* = 5) (Figure 7(a)). LPS also strongly increased AP-1 activation by 9.6- and 25.3-fold, respectively, while AS-IV treatment significantly diminished LPS-induced AP-1 activity by 41% and 49%, respectively, in both lung and heart (*P* < 0.05, *n* = 5) (Figure 7(b)).

Our data provide new evidence that the inhibitory effect of AS-IV on LPS-induced inflammatory gene expression is mainly through modulating NF-*κ*B and AP-1 DNA-binding activity. These data indicate that the ability of AS-IV to suppress the NF-*κ*B and AP-1 pathways is the major underlying mechanism contributing to its anti-inflammatory potential* in vivo*. It is well documented that the “classic” TLR4 pathway plays a major role in LPS signaling during sepsis [36]. TLR4 recognizes LPS from Gram-negative bacteria and mediates the innate immune response by activating I*κ*B kinase (IKK) and mitogen-activated protein kinase kinases (MKK), which in turn activate NF-*κ*B and AP-1, respectively [5, 36]. Our results further show that AS-IV inhibits LPS-induced* TLR4* gene expression in lung and heart by 30% and 65%, respectively (Figures 2 and 4), which is consistent with its inhibitory effects on NF-*κ*B and AP-1 activation in the same tissues. These data suggest that the inhibition of* TLR4* expression might be one of the mechanisms by which AS-IV affects the upstream targets of these pathways. Some* in vitro* studies have shown that AS-IV activates the PI3K/Akt pathway [29], which is known to negatively regulate LPS-induced acute inflammatory responses by modulating the NF-*κ*B and AP-1 pathways [9, 37, 38]. Therefore, PI3K/Akt activation may be one of the underlying mechanisms for the anti-inflammatory activity of AS-IV. Further, as a novel antioxidant [39, 40], AS-IV may modulate LPS-induced formation of reactive oxygen species and subsequent activation of the redox-sensitive NF-*κ*B and AP-1 pathways at different levels [40]. However, all these mechanisms observed* in vitro* need to be further investigated* in vivo.*

A limitation of our study is that we had to apply AS-IV by i.p. injection because it is poorly bioavailable. Previous studies on dogs and rats have found that only 7.4% and 3.7%, respectively, of orally supplemented AS-IV were absorbed [41, 42]. This is mainly due to AS-IV's low fat solubility and low transmittance in the small intestine [43]. In fact, in Chinese medicine AS-IV is clinically used as intravenous (i.v.) therapy. Upon i.v. injection of AS-IV at 0.75 mg/kg in rats and 0.5 mg/kg in dogs, the maximum plasma concentrations reached were 3.79 *µ*g/mL and 4.39 *µ*g/mL, respectively, and the elimination half-life (*t*_1/2_) was 98 min and 60 min, respectively [44, 45]. The highest concentration was found in lung and liver tissues (2.8–2.9 *µ*g/g) after i.v. injection of 1.5 mg/kg AS-IV, whereas heart, muscle, skin, and kidney contained moderate amounts (0.16–1.0 *µ*g/g) [45]. As* Astragalus* and AS-IV are now widely used in Chinese medicine and are also available in Europe and the US as dietary supplements, chemical modifications to improve the absolute bioavailability of AS-IV while maintaining its biological activity could be a focus of future research.

## 4. Conclusion

In conclusion, our data provide new evidence that AS-IV inhibits LPS-induced acute inflammatory responses* in vivo* by modulating the NF-*κ*B and AP-1 signaling pathways. Our results might lead to the identification of AS-IV as a natural compound or chemically derived drug that may be useful for the prevention or treatment of inflammatory diseases. We believe that the molecular basis for its therapeutic efficacy is intriguing and warrants further investigation.

## Abbreviations

AP-1: Activator protein-1
AS-IV: Astragaloside IV
b.w.: Body weight
GAPDH: Glyceraldehyde-3-phosphate dehydrogenase
ICAM-1: Intercellular adhesion molecule-1
i.p.: Intraperitoneal
i.v.: Intravenous
IKK: I*κ*B kinase
IL: Interleukin
LPS: Lipopolysaccharide
MCP-1: Monocyte chemoattractant protein-1
MPO: Myeloperoxidase
NF-*κ*B: Nuclear factor *κ*B
PMN: Polymorphonuclear neutrophils
Redox: Reduction-oxidation
ROS: Reactive oxygen species
sICAM-1: Soluble intercellular adhesion molecule-1
sVCAM-1: Soluble vascular cell adhesion molecule-1
TLR4: Toll-like receptor-4
TNF*α*: Tumor necrosis factor *α*
VCAM-1: Vascular cell adhesion molecule-1.

## References

[1] Aird W. C. (2003). The role of the endothelium in severe sepsis and multiple organ dysfunction syndrome. *Blood*.

[2] Parrillo J. E. (1993). Pathogenetic mechanisms of septic shock. *The New England Journal of Medicine*.

[3] Remick D. G. (2007). Pathophysiology of sepsis. *The American Journal of Pathology*.

[4] Collins T., Read M. A., Neish A. S., Whitley M. Z., Thanos D., Maniatis T. (1995). Transcriptional regulation of endothelial cell adhesion molecules: NF-*κ*B and cytokine-inducible enhancers. *The FASEB Journal*.

[5] Liu S. F., Malik A. B. (2006). NF-*κ*B activation as a pathological mechanism of septic shock and inflammation. *American Journal of Physiology: Lung Cellular and Molecular Physiology*.

[6] Zhang W.-J., Hufnagl P., Binder B. R., Wojta J. (2003). Antiinflammatory activity of astragaloside IV is mediated by inhibition of NF-*κ*B activation and adhesion molecule expression. *Thrombosis and Haemostasis*.

[7] Weber C., Erl W., Pietsch A., Strobel M., Ziegler-Heitbrock H. W. L., Weber P. C. (1994). Antioxidants inhibit monocyte adhesion by suppressing nuclear factor-*κ*B mobilization and induction of vascular cell adhesion molecule-1 in endothelial cells stimulated to generate radicals. *Arteriosclerosis and Thrombosis*.

[8] Zhang W.-J., Frei B. (2001). *α*-Lipoic acid inhibits TNF-*α*-induced NF-*κ*B activation and adhesion molecule expression in human aortic endothelial cells. *The FASEB Journal*.

[9] Zhang W. J., Wei H., Hagen T., Frei B. (2007). *α*-Lipoic acid attenuates LPS-induced inflammatory responses by activating the phosphoinositide 3-kinase/Akt signaling pathway. *Proceedings of the National Academy of Sciences of the United States of America*.

[10] Chu C., Qi L.-W., Liu E.-H., Li B., Gao W., Li P. (2010). Radix astragali (Astragalus): latest advancements and trends in chemistry, analysis, pharmacology and pharmacokinetics. *Current Organic Chemistry*.

[11] Cho W. C. S., Leung K. N. (2007). *In vitro* and *in vivo* immunomodulating and immunorestorative effects of *Astragalus membranaceus*. *Journal of Ethnopharmacology*.

[12] Denzler K. L., Waters R., Jacobs B. L., Rochon Y., Langland J. O. (2010). Regulation of inflammatory gene expression in PBMCs by immunostimulatory botanicals. *PloS ONE*.

[13] Chen X. J., Bian Z.-P., Lu S. (2006). Cardiac protective effect of Astragalus on viral myocarditis mice: comparison with Perindopril. *The American Journal of Chinese Medicine*.

[14] Xu X.-L., Ji H., Gu S.-Y., Shao Q., Huang Q.-J., Cheng Y.-P. (2008). Cardioprotective effects of Astragali Radix against isoproterenol-induced myocardial injury in rats and its possible mechanism. *Phytotherapy Research*.

[15] Fu S., Zhang J., Menniti-Ippolito F. (2011). Huangqi injection (a traditional chinese patent medicine) for chronic heart failure: a systematic review. *PLoS ONE*.

[16] Ryu M., Kim E. H., Chun M. (2008). Astragali Radix elicits anti-inflammation via activation of MKP-1, concomitant with attenuation of p38 and Erk. *Journal of Ethnopharmacology*.

[17] Gao X.-H., Xu X.-X., Pan R. (2009). Saponin fraction from *Astragalus membranaceus* roots protects mice against polymicrobial sepsis induced by cecal ligation and puncture by inhibiting inflammation and upregulating protein C pathway. *Journal of Natural Medicines*.

[18] Jiang J. B., Qiu J. D., Yang L. H., He J. P., Smith G. W., Li H. Q. (2010). Therapeutic effects of astragalus polysaccharides on inflammation and synovial apoptosis in rats with adjuvant-induced arthritis. *International Journal of Rheumatic Diseases*.

[19] Gui S.-Y., Wei W., Wang H. (2006). Effects and mechanisms of crude astragalosides fraction on liver fibrosis in rats. *Journal of Ethnopharmacology*.

[20] Wang S., Li J., Huang H. (2009). Anti-hepatitis B virus activities of astragaloside IV isolated from *Radix Astragali*. *Biological and Pharmaceutical Bulletin*.

[21] Li M., Wang W., Xue J., Gu Y., Lin S. (2011). Meta-analysis of the clinical value of Astragalus membranaceus in diabetic nephropathy. *Journal of Ethnopharmacology*.

[22] Cho W. C. S., Leung K. N. (2007). *In vitro* and *in vivo* anti-tumor effects of *Astragalus membranaceus*. *Cancer Letters*.

[23] Tohda C., Tamura T., Matsuyama S., Komatsu K. (2006). Promotion of axonal maturation and prevention of memory loss in mice by extracts of Astragalus mongholicus. *British Journal of Pharmacology*.

[24] Song J.-Z., Yiu H. H. W., Qiao C.-F., Han Q.-B., Xu H.-X. (2008). Chemical comparison and classification of Radix Astragali by determination of isoflavonoids and astragalosides. *Journal of Pharmaceutical and Biomedical Analysis*.

[25] Kitagawa I., Wang H. K., Saito M., Takagi A., Yoshikawa M. (1983). Saponin and sapogenol. XXXV. Chemical constituents of astragali radix, the root of Astragalus membranaceus Bunge. 2. Astragalosides I, II, and IV, acetylastragaloside I and isoastragaloside I and II. *Chemical and Pharmaceutical Bulletin*.

[26] Zhang W.-D., Zhang C., Wang X. H. (2006). Astragaloside IV from *Astragalus membranaceus* shows cardioprotection during myocardial ischemia *in vivo* and *in vitro*. *Planta Medica*.

[27] Xu X.-L., Ji H., Gu S.-Y., Shao Q., Huang Q.-J., Cheng Y.-P. (2007). Modification of alterations in cardiac function and sarcoplasmic reticulum by astragaloside IV in myocardial injury *in vivo*. *European Journal of Pharmacology*.

[28] Zhang W. J., Wojta J., Binder B. R. (1997). Regulation of the fibrinolytic potential of cultured human umbilical vein endothelial cells: astragaloside IV downregulates plasminogen activator inhibitor-1 and upregulates tissue-type plasminogen activator expression. *Journal of Vascular Research*.

[29] Zhang L., Liu Q., Lu L., Zhao X., Gao X., Wang Y. (2011). Astragaloside IV stimulates angiogenesis and increases hypoxia-inducible factor-1*α* accumulation via phosphatidylinositol 3-kinase/akt pathway. *Journal of Pharmacology and Experimental Therapeutics*.

[30] Zhao J., Yang P., Li F. (2012). Therapeutic effects of astragaloside IV on myocardial injuries: multi-target identification and network analysis. *PLoS ONE*.

[31] Zhang W. J., Wei H., Frei B. (2009). Genetic deficiency of NADPH oxidase does not diminish, but rather enhances, LPS-induced acute inflammatory responses in vivo. *Free Radical Biology and Medicine*.

[32] Awad S. S. (2003). State-of-the-art therapy for severe sepsis and multisystem organ dysfunction. *The American Journal of Surgery*.

[33] Zhang W.-J., Wei H., Tien Y.-T., Frei B. (2011). Genetic ablation of phagocytic NADPH oxidase in mice limits TNF*α*-induced inflammation in the lungs but not other tissues. *Free Radical Biology and Medicine*.

[34] Juskewitch J. E., Platt J. L., Knudsen B. E., Knutson K. L., Brunn G. J., Grande J. P. (2012). Disparate roles of marrow-and parenchymal cell-derived TLR4 signaling in murine LPS-induced systemic inflammation. *Scientific Reports*.

[35] Böhrer H., Qiu F., Zimmermann T. (1997). Role of NF*κ*B in the mortality of sepsis. *The Journal of Clinical Investigation*.

[36] Pålsson-McDermott E. M., O'Neill L. A. J. (2004). Signal transduction by the lipopolysaccharide receptor, Toll-like receptor-4. *Immunology*.

[37] Schabbauer G., Tencati M., Pedersen B., Pawlinski R., Mackman N. (2004). PI3K-Akt pathway suppresses coagulation and inflammation in endotoxemic mice. *Arteriosclerosis, Thrombosis, and Vascular Biology*.

[38] Williams D. L., Li C., Ha T. (2004). Modulation of the phosphoinositide 3-kinase pathway alters innate resistance to polymicrobial sepsis. *Journal of Immunology*.

[39] Gui D., Guo Y., Wang F. (2012). Astragaloside IV, a novel antioxidant, prevents glucose-induced podocyte apoptosis in vitro and in vivo. *PLoS ONE*.

[40] He Y., Du M., Gao Y. (2013). Astragaloside IV attenuates experimental autoimmune encephalomyelitis of mice by counteracting oxidative stress at multiple levels. *PLoS ONE*.

[41] Zhang Q., Zhu L.-L., Chen G.-G., Du Y. (2007). Pharmacokinetics of astragaloside*iv* in beagle dogs. *European Journal of Drug Metabolism and Pharmacokinetics*.

[42] Du Y., Zhang Q., Chen G. G., Wei P., Tu C. Y. (2005). Pharmacokinetics of Astragaloside IV in rats by liquid chromatography coupled with tandem mass spectrometry. *European Journal of Drug Metabolism and Pharmacokinetics*.

[43] Huang C. R., Wang G. J., Wu X. L. (2006). Absorption enhancement study of astragaloside IV based on its transport mechanism in Caco-2 cells. *European Journal of Drug Metabolism and Pharmacokinetics*.

[44] Gu Y., Wang G., Pan G., Fawcett J. P., A J., Sun J. (2004). Transport and bioavailability studies of astragaloside IV, an active ingredient in Radix astragali. *Basic and Clinical Pharmacology and Toxicology*.

[45] Zhang W. D., Zhang C., Liu R.-H. (2006). Preclinical pharmacokinetics and tissue distribution of a natural cardioprotective agent astragaloside IV in rats and dogs. *Life Sciences*.

